# Investigation on the Strengthening Mechanisms of Nickel Matrix Nanocomposites

**DOI:** 10.3390/nano11061426

**Published:** 2021-05-28

**Authors:** Íris Carneiro, José Valdemar Fernandes, Sónia Simões

**Affiliations:** 1 DEMM, Department of Metallurgical and Materials Engineering, University of Porto, Rua Dr. Roberto Frias, 4200-465 Porto, Portugal; up201207199@fe.up.pt; 2 LAETA/INEGI, Institute of Science and Innovation in Mechanical and Industrial Engineering, Rua Dr. Roberto Frias, 4200-465 Porto, Portugal; 3 Centre for Mechanical Engineering, Materials and Processes, (CEMMPRE), Department of Mechanical Engineering, University of Coimbra, Rua Luís Reis Santos, Pinhal de Marrocos, 3030-788 Coimbra, Portugal; valdemar.fernandes@dem.uc.pt

**Keywords:** strengthening mechanisms, carbon nanotubes, metal matrix nanocomposites, powder metallurgy, load transfer, second phase particles, dislocation density, grain size

## Abstract

The strengthening effect of carbon nanotubes (CNTs) in metal matrix nanocomposites occurs due to several mechanisms that act simultaneously. The possible strengthening mechanisms for metal matrix nanocomposites reinforced with CNTs consist of: (1) load transfer, (2) grain refinement and texture strengthening, (3) second phase strengthening, and (4) strain hardening. The main focus of this work is to identify the strengthening mechanisms that play a role in the case of the Ni-CNT nanocomposite produced by powder metallurgy. For the dispersion and mixing of the metallic powders with CNTs, two different routes were performed by ultrasonication and ball milling. The results indicated that four different strengthening mechanisms are present in the nanocomposites and had a different contribution to the final mechanical properties. The load transfer and the increase in dislocation density seem to strongly affect the properties and microstructure of the nanocomposite. The grain refinement and the presence of second phase particles have a small contribution in the strengthening of this nanocomposite, since the introduction of CNTs in the Ni matrix slightly affects the size and orientation of the grains in the matrix and a few nanometric particles of Ni_3_C were identified.

## 1. Introduction

Nowadays, there is a great interest in developing more sustainable mobility, which can be achieved by reducing fuel consumption and, consequently, emissions from the transport industry. In this sense, the scientific community has been dedicated to studying and developing high-performance lightweight alloys to produce structural components. Metal matrix composites emerge as great candidates to face the challenge of producing these lightweight materials [1,2]. The use of nanometric reinforcements to produce metallic matrix nanocomposites (MMNCs) is one of the possible ways to effectively overcome the existing limitations and produce materials with a unique combination of mechanical properties [3,4,5]. Different types of materials are used as reinforcement metal matrix composites, such as ceramic, intermetallic, carbides, and carbon-based nanomaterials. These reinforcements can be used at the nanoscale, which has shown greater efficiency in reinforcing metals instead of micrometric reinforcements. Within this range of nano-sized reinforcements, carbon-based nanoparticles, such as carbon nanotubes (CNTs), have been the focus of intense interest in producing MMNCs [6,7,8,9,10,11,12,13,14,15,16].

The reinforcement effect occurs due to several strengthening mechanisms that act simultaneously and, therefore, its identification presents several difficulties. For this reason, the potential of these nanocomposites can be compromised by the lack of scientific knowledge regarding the individual identification of the strengthening mechanisms and the effect of the presence of CNTs in the microstructure, which determines the MMNCs properties. The possible strengthening mechanisms for metal matrix nanocomposites reinforced with CNTs consist of: (1) load transfer, (2) grain refinement and texture strengthening, (3) second phase strengthening, and (4) strain hardening.

The load transfer mechanism consists of transferring the strength from the matrix to the CNTs since the reinforcement has higher mechanical properties. This is the most widely studied and frequently mentioned mechanism for nanocomposites [16,17,18,19,20,21,22,23]. For the load transfer to occur efficiently, it is necessary to ensure that the reinforcement is homogeneously dispersed in the metallic matrix and has a strong interface bonding. The formation of second phase particles, resulting from the reaction between the CNTs and the matrix at the matrix-reinforcement interface, improves their bond and, consequently, enhances the load transfer mechanism [17,18,19]. The occurrence of this mechanism is often evaluated by observing the fracture surface of nanocomposites and analyzing the presence of pulled-out or fractured CNTs, which means that the load was transferred effectively [20,21]. Some authors used in situ tensile tests to study this mechanism [22,23]. Boels et al. [22], performed in-situ tensile tests, by SEM, of Al and Al-CNT to identify the load transfer mechanism. SEM images of the nanocomposites revealed stretched CNTs and pull-out failure across the fracture surface. Chen et al. [23], also investigated the fracture of CNTs during the in-situ tensile tests of Al-CNT nanocomposites in SEM equipment. The authors observed that the CNTs seemed to act like a bridge, restricting the growth of crack nucleated at the Al matrix. With the increase of the tensile stress, the CNT started to fracture. Multiple stages of the peeling fracture process were observed: (1) the CNT fractures initially at the outer layer and the wall-breaking grows from the outer walls to the inner walls, under the load transferred to the CNT walls; (2) the fracture position is axially shifted to another position on the CNT wall; and (3) the peeling behavior continues until all the walls are fractured. 

The grain refinement, which can be observed in nanocomposites, can contribute to the strengthening mechanisms, since the decrease in grain size and the consequent increase in boundary surface increases the resistance to plastic deformation, as illustrated by the Hall-Petch equation. The CNTs significantly affect the grain boundary migration during the production process, leading to grain refinement of the metallic matrix through Zener pinning. Xu et al. [24] observed the grain refinement for the Al-CNTs nanocomposites produced by powder metallurgy, using ball milling as a dispersion technique following hot-extrusion. The nanocomposites revealed a microstructure characterized by smaller grains than the sample of Al processed under the same conditions. The authors state that during sintering, the CNTs have a pinning effect on the grain boundaries. This mechanism can also be observed when the reaction of the reinforcement with the metallic matrices promotes the formation of nanometric phases. The presence of second phase particles can also act as occurs with CNTs, promoting grain refinement due to the pinning effect. Besides, the formation of these particles can improve the bonding between the CNTs and the matrix, as reported by some works [24,25,26,27]. 

Strain hardening due to the presence of CNTs in the metallic matrix can also assist in strengthening the nanocomposites. Orowan strengthening occurs when the reinforcement affects the dislocation motion, and, strengthening due to thermal mismatch leads to the promotion of geometrically necessary dislocation due to thermal mismatch between the matrix and the CNTs. The presence of a second phase can also contribute to this mechanism. These particles can also act as obstacles to the rearrangement of dislocations and affect the nanocomposite mechanical properties [28]. 

The main objective of this work is to investigate the strengthening mechanism in Ni-CNT nanocomposites produced by powder metallurgy. Since the dispersion of the reinforcement in the matrix is one of the most important parameters to obtain an effective reinforcement of the nanocomposites, the effect of using two different dispersion and mixing routes was evaluated in this work. Microstructural characterization by scanning electron microscopy (SEM), electron backscatter diffraction (EBSD), and high-resolution transmission electron microscopy (HRTEM), were conducted to identify the strengthening mechanisms that play a role in these nanocomposites. 

## 2. Materials and Methods

The production of Ni-CNT (1.00% vol.) nanocomposite was performed by a classic route of powder metallurgy. Nickel powders and multi-walled carbon nanotubes (MWCNTs) were used. The nickel powders exhibit a D_50_ of 15 µm and a maximum powder size of 60 µm with a purity of 99.5% were provided by Goodfellow Cambridge Ltd. (Huntingdon, UK), while the MWCNTs were provided by Fibermax Nanocomposites Ltd. (London, UK). Both powders used in this work, nickel, and MWCNTs, were fully characterized in previous works [7,8,29], including the microstructure of nickel powder particles as-received and during the production of nanocomposites.

**Figure 1:**
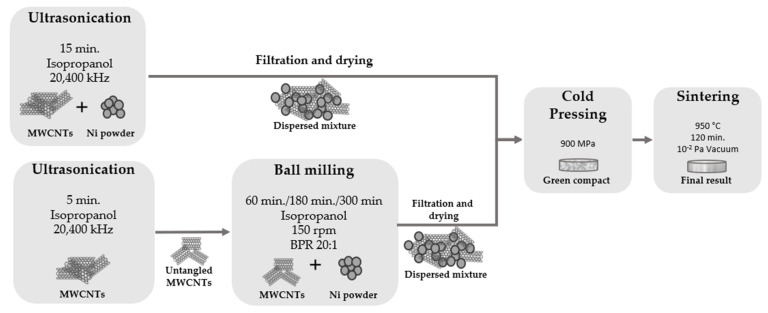
Schematic flowchart of the different routes used on the production of Ni-CNT nanocomposites.

To effectively produce this type of nanocomposites, and most importantly, for efficient reinforcement and strengthening mechanisms to occur, the dispersion of MWCNTs in the metallic matrix must be uniform, which makes the dispersion/mixture step of the production route crucial to obtain the desired result. In the present work, the mixture and dispersion of the powders were performed by two different techniques: ultrasonication and ultrasonication combined with ball milling, as schematized in Figure 1. Firstly, the ultrasonication technique was used for the dispersion/mixture step of the MWCNTs and Ni particles using the best conditions (15 min in isopropanol) previously reported [7,8,29]. On a second route, the ultrasonication method is used in the same conditions but only for a short time (5 min) to untangle some of the agglomerated MWCNTs, followed by ball milling to disperse and mix the untangled MWCNTs with the nickel powders. This method was performed with a ball-to-powder ratio of 20:1, using 150 rpm with different times of 60 min, 180 min, and 300 min.

To identify the strengthening mechanisms of the nanocomposites, the use and combination of several advanced characterization techniques, such as electron backscatter diffraction (EBSD) and high-resolution transmission electron microscopy (HRTEM), is crucial.

Initially, optical microscopy (OM) combined with image analysis software was used to overview the as-produced samples and to quantify the percentage of pores and agglomerates in each sample. To achieve this objective, a DM 4000 M optical microscope equipped with a Leica DFC 420 camera (Leica Microsystems, Wetzlar, Germany) was used and the image analysis quantification was performed with Leica Application Suite software (Leica Microsystems, Wetzlar, Germany). The average grain value was obtained measuring up to 300 grains per sample from OM imagens and EBSD grain size maps. Raman spectroscopy experiments (Horiba Scientific, Kyoto, Japan) were conducted with a 442 nm wavelength laser to evaluate the damage and defects induced in the CNT during dispersion and mixture processes. 

The mechanical characterization was also performed to understand the effect of the reinforcement on the Ni matrix. In this sense, Vickers microhardness tests were performed using a Duramin-1 durometer (Duramin-1; Struers A/S, Ballerup, Denmark), which was used to make about 12 indentations in each sample, with a load of 1.961 N.

Since EBSD is a complete technique with great potential to study microstructural features in great detail, the samples produced were analyzed using an SEM high-resolution FEI QUANTA 400 FEG SEM equipment (FEI Company, Hillsboro, OR, USA) accoupled with an EBSD detector TSL-EDAX EBSD Unit (EDAX Inc. (Ametek), Mahwah, NJ, USA)). The obtained raw data are submitted to a routine dilatation clean-up, where the grain tolerance angle and minimum grain size of 15° and 2 points, respectively, are determined, which is essential to prevent doubtful results; only then, the data are used to elaborate a wide range of maps and graphs using TSL OIM Analysis 5.2. The overall EBSD maps- inverse pole figures (IPFs), kernel average misorientation (KAM), and grain orientation spread (GOS) were elaborated as described, with conditions similar to those of previous work [29]. 

The IPF maps illustrate the crystallographic orientation in each point, making it possible to briefly study the occurrence of preferential grain orientation, distinguishing between the grains giving the perception of the grain size, and have general information of misorientation existing within each grain and a general idea of the crystallographic orientation. GOS maps are also fundamentally based on grain orientation, though with different principles. These maps focus on the deviation between the average orientation of each grain the surrounding points, which make them suitable to investigate the recovery and recrystallization processes [30,31,32,33]. In GOS maps, by definition, the recrystallized grains are the ones showing an orientation spread until 1°, according to Bair et al. [33]. The dislocation density was mainly evaluated by KAM maps and misorientation graphs, representing the misorientation between each point and the neighbor points. In addition, image quality (IQ) maps were superimposed not only with the delineation of low-angle boundaries (typically 5° and 15°), often associated with dislocation cells, high-angle boundaries (15° and 180°) that generally represent grain boundaries, and also the most common and highly influential nickel twins (Σ3 and Σ9) [33,34]. Distribution graphs of grain boundary character were also elaborated to study in detail the influence of the addition of CNTs and the processing conditions on the formation of each type of boundary. Geometrically necessary dislocation (GND) densities were estimated using ATEX software [35]. 

Differential scanning calorimetry (DSC, Setaram Kep Technologies, Caluire-et-Cuire, France) performed, from room temperature up to 1000 °C, with heating rate of 5 °C min^−1^ in an argon atmosphere.

Second phase particle formation was investigated by X-ray diffraction (XRD) technique, with Panalytical X’Pert Pro MPD equipment (Malvern Panalytical Ltd., Malvern, UK) using CuKα radiation and collect patterns from 20° to 100° (2*θ*) in a *θ*-2*θ* Bragg-Brentano mode.

The use of high-resolution TEM was beneficial for the detailed study of the bonding of CNTs in the metallic matrix and the formation of second phase particles due to the CNT reaction with the matrix. For this reason, a high-resolution TEM (HRTEM) (FEI Company, Hillsboro, OR, USA) was used for this purpose.

## 3. Results and Discussion

The microstructural characterization results reveal that all nanocomposites exhibit a microstructure consisting of equiaxed grains with CNTs agglomerates, mainly located in the grain boundaries. 

**Figure 2:**
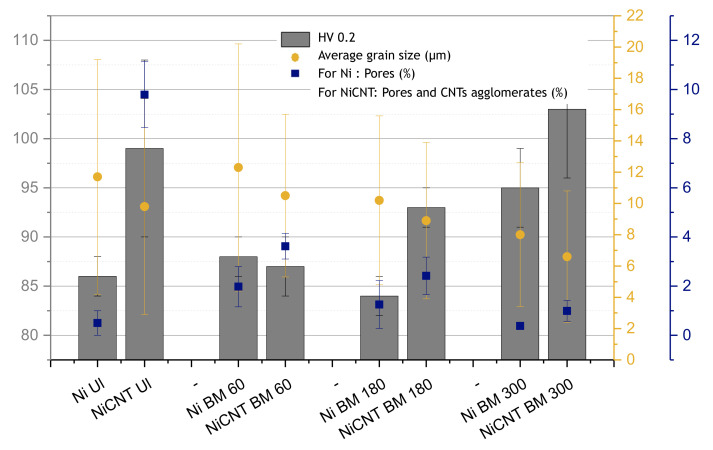
Hardness values (HV 0.2), average grain size, and number of pores and CNTs agglomerates for the samples produced with different dispersion/mixture processes: ultrasonication (Ul) and ball milling (BM) for 60, 180, and 300 min.

Figure 2 shows the hardness values (HV 0.2), the average grain size, and the percentage of pores and CNTs agglomerates for the samples produced using different dispersion and mixing techniques, with and without reinforcement. The dispersion and mixture processes, namely ultrasonication (UL) and ball milling (BM) for different times (60, 180, and 300 min), were applied to both Ni and Ni-CNT for comparison purposes.

The nanocomposites produced by ultrasonication as dispersion/mixture process showed a greater increase in hardness than the sample produced under the same conditions without the reinforcement. For nanocomposites produced through CNTs untangle by ultrasonication and dispersion/mixture by ball milling, the increase in the hardness is only observed for the nanocomposites produced by ball milling with a time of more than 60 min. The sample produced with 60 min ball milling shows a hardness value similar to the sample without reinforcement. This can probably be explained by the poor dispersion of reinforcement due to insufficient time of the dispersion process used, not leading to the expected strengthening effect. Nanocomposites produced with ultrasonication or ball milling for 180 min as dispersion/mixture process present a greater increase in hardness, revealing to be the best conditions for the production of these composites. That is, the dispersion of reinforcement is one of the main aspects of success in the production of nanocomposites. 

The dispersion of CNTs can be evaluated by the amount of the pores and CNTs clusters. Figure 2 reveals that all nanocomposites have a high number of pores and CNTs agglomerates. The presence of pores characterizes the samples produced by powder metallurgy. For the nanocomposites, SEM images, not shown here, reveal that CNTs fill the pores; nevertheless, it is not guaranteed that all pores are filled. For this reason, the number of pores and CNTs clusters was measured together. The samples produced by the ultrasonication dispersion technique have the most significant porosity. The decrease in porosity can be achieved through ball milling as a process of dispersion and mixing. However, ball milling times longer than 60 min are required. It should be noted that although the reduction in the percentage of pores with this dispersion/mixture process is effective, the results reveal that the most significant increase in hardness is observed for nanocomposites with the largest number of pores (produced using ultrasonication as dispersion/mixture process). This can be related to the damage suffered by CNTs during the production process. Raman spectroscopy results revealed that for the nanocomposites produced by ball milling a significant increase in the ratio of the intensity of the D band (ID) to the intensity of the G band (IG) is observed (from ID/IG of 1.03 to the ones produced by ultrasonication to 1.58 for the nanocomposites produced by ball milling). The ball milling promotes more significant damage to the structure of CNTs, reducing their potential for strengthening. For effective strengthening, uniform dispersion of the CNTs and strong bonding between the CNTs and the matrix are required, but also a structure of CNTs without damage. 

**Figure 3:**
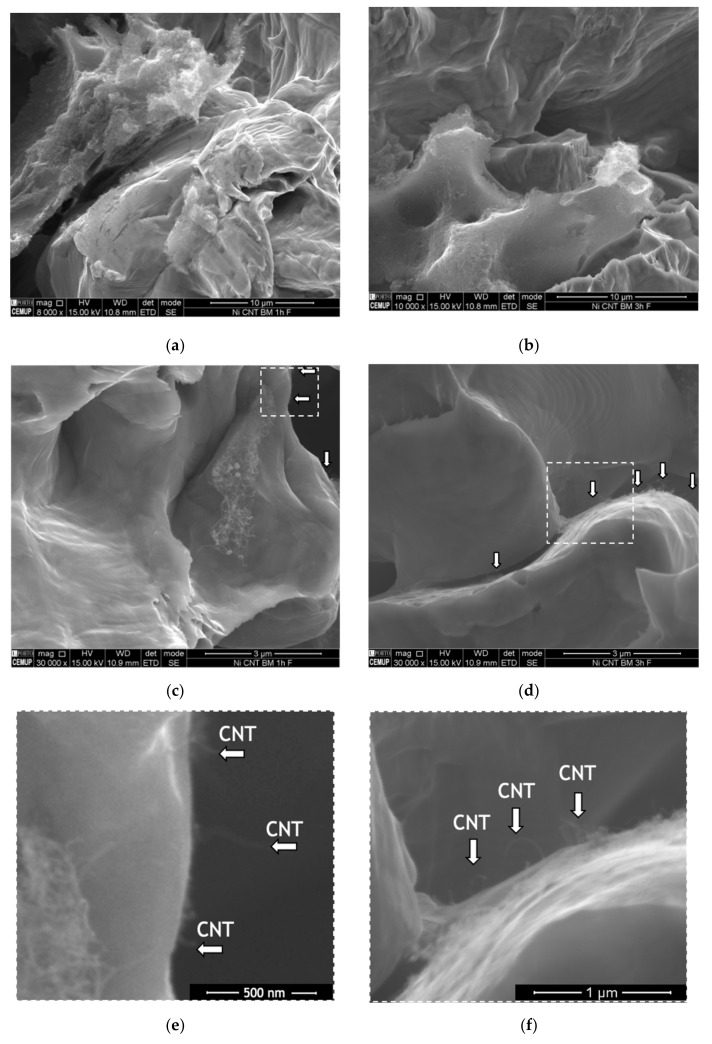
Scanning electron microscopy (SEM) images of the fracture surface of the nanocomposites produced using ball milling for (**a**,**c**,**e**) 60 min; and (**b**,**d**,**f**) 180 min.

Fracture surfaces of the nanocomposites were observed in order to investigate the load transfer mechanism. Figure 3 shows examples of SEM images of the fracture surface of the nanocomposites produced by ball milling during 60 and 180 min. 

The low magnification image shows that the nanocomposite produced using a short ball milling time exhibit more and larger CNTs agglomerates than those observed on the rupture surface of the nanocomposite produced with 180 min of ball milling. This observation confirms the hardness results, proving that it is necessary more time than 60 min to improve the dispersion of the CNTs in the matrix. The poor dispersion promotes a less effective strengthening of the matrix, resulting in a lower hardness value. In contrast, CNTs well-dispersed in the matrix are suitable for the load transfer strengthening mechanism since leads to an increase in the CNT embedded in the matrix leading to a better bond of the CNT/matrix interface. High magnification SEM images show (see arrows in the figures) that fractured or pulled-out CNTs can be identified at the grain boundaries. These observations prove that load transfer is one of the strengthening mechanisms present. When the matrix undergoes plastic deformation, nucleation and crack propagation occur. CNTs act as bridges and restrain crack growth. 

The load transfer mechanism is commonly referred to as the one that most contributes to the strengthening effect in nanocomposites reinforced with CNTs [16,19,22,23]. However, other mechanisms are simultaneously present, playing a role in increasing the mechanical properties of the nanocomposites. Microstructural features as grain size, grain orientation, texture, and presence of second phase particles can also contribute to the strengthening of the nanocomposites [13,14,24]. Figure 2 revealed that all nanocomposites exhibit a slightly smaller grain size than the sample without the reinforcement. The dispersion technique does not appear to have a significant effect on the grain refinement, unlike the effect of the presence of CNTs. However, for long ball milling times, the samples have smaller grain sizes. This is related to the plastic deformation that occurs during this process.

**Figure 4:**
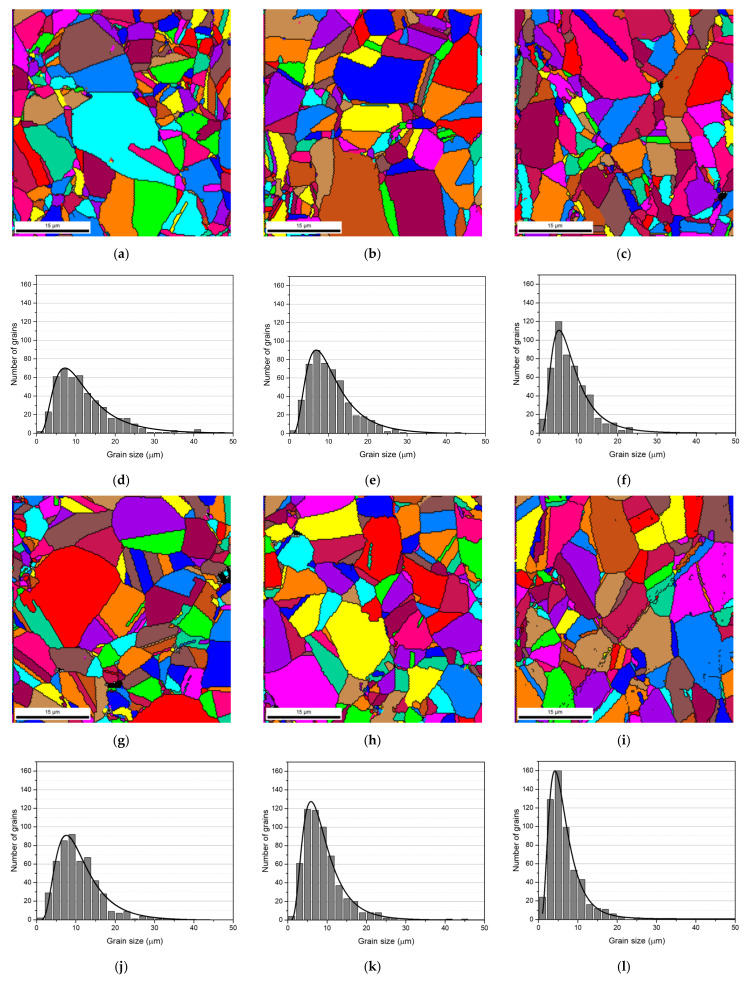
Grain size maps and distributions of Ni produced by ball milling for (**a**,**d**) 60 min, (**b**,**e**) 180 min, and (**c**,**f**) 300 min, and of Ni-CNT produced with (**g**,**j**) 60 min, (**h**,**k**) 180 min, and (**i**,**l**) 300 min.

The grain refinement of the nanocomposites was investigated by EBSD. Figure 4 present the grain size maps and grain size distributions for the Ni and Ni-CNT nanocomposites produced with 60, 180, and 300 min of ball milling. Based on these results, it is clear that the nanocomposites are characterized by smaller grains than the Ni samples produced under the same conditions, mainly close to the CNTs clusters. The grain orientation of the nanocomposites is also an important characteristic that needs to be analyzed. 

**Figure 5:**
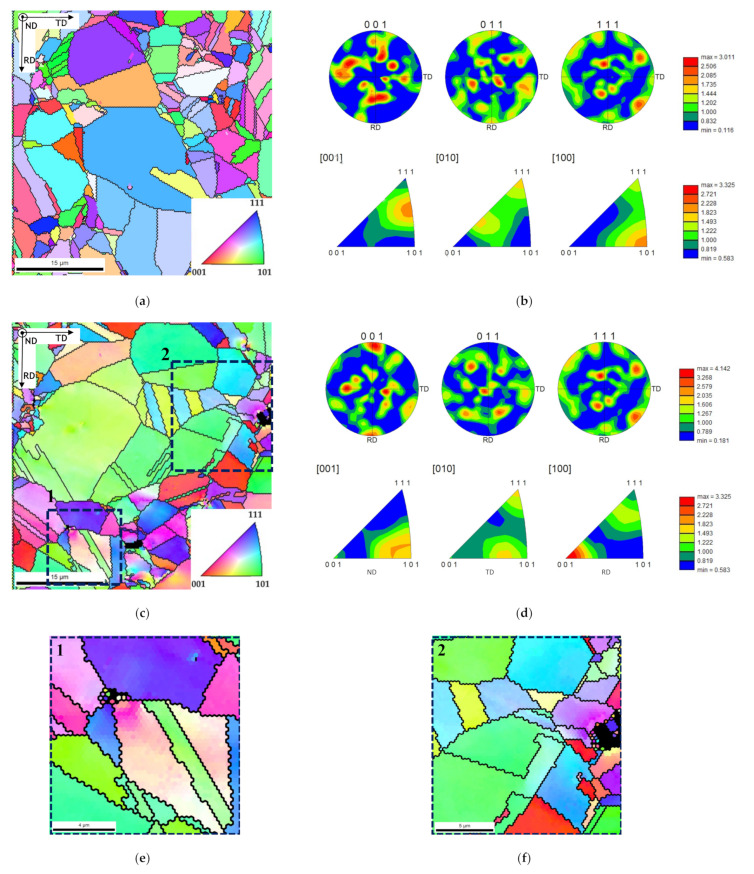
IPF maps and IPF and PF charts of samples produced by ball milling for 60 min: (**a**,**b**) Ni; (**c**,**d**) Ni-CNT; and (**e**,**f**) high magnification of regions marked as 1 and 2 areas in (**c**).

Figure 5 shows the IPF maps and charts revealing that the grain orientation of the matrix changes in the presence of CNTs. The nanocomposites exhibited a different texture than the samples without reinforcement, for example, with respect to the plane parallel to the sample surface (i.e., perpendicular to ND), which are, respectively, the (101) and the (212) planes. This change in the grain orientation was also observed for the Ni nanocomposites produced by ultrasonication [29]. In addition, in the case of nanocomposites, the grains close to the CNTs agglomerates reveal a different crystal orientation of other larger grains apparently without CNTs. The introduction of the CNTs on the Ni matrix may affect the grain rotation and grow during the sintering resulting in a different crystal orientation close to the CNT clusters. 

**Figure 6:**
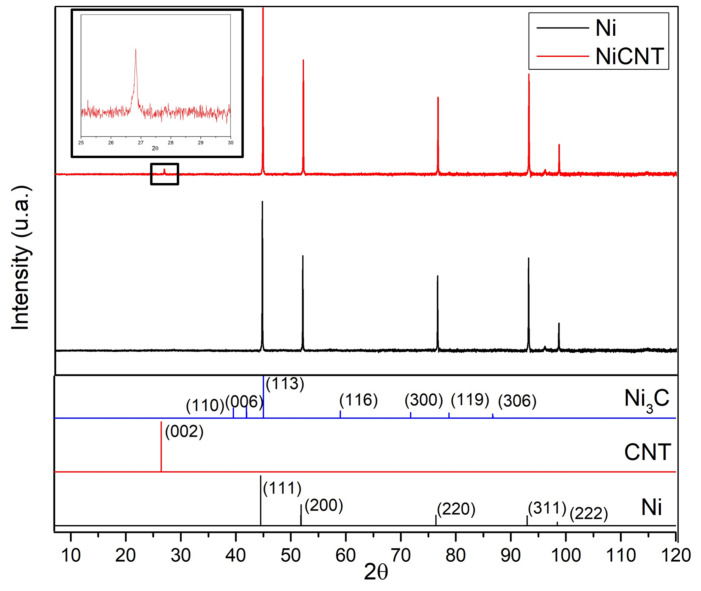
XRD patterns of Ni and Ni-CNT nanocomposite and XRD peaks for, Ni, CNTs, and Ni_3_C phases.

**Figure 7:**
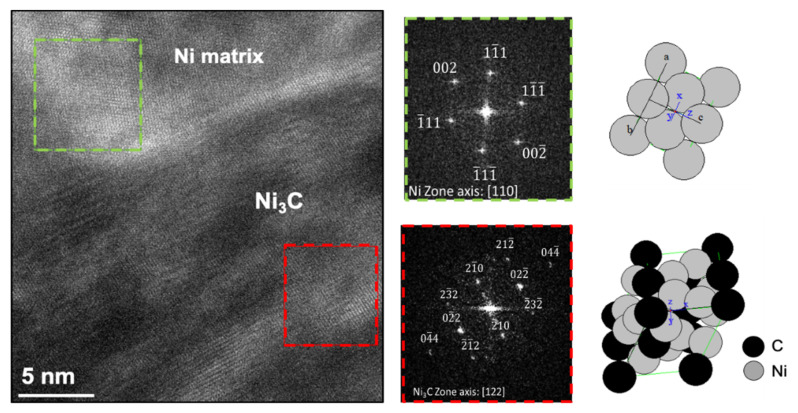
HRTEM image of Ni-CNT nanocomposite and FFT images of the two marked regions, indicating the presence of second phase particles indexed as Ni_3_C.

XRD investigations were performed in order to identify the presence of second phase particles in the samples. Figure 6 shows the XRD patterns for Ni and Ni-CNT samples. These results show that the only difference between the samples is the presence of a C peak that corresponds to (002) planes of the CNTs, in the case of nanocomposites. This peak confirms the presence of the CNTs in the nanocomposite samples. The Ni_3_C was not detected by this characterization technique. This may mean that this phase was not formed or is formed, but to a small extent at the nanoscale. HRTEM images led to a more detailed investigation of the second phase formation. Figure 7 reveals that Ni_3_C, can be identified in few regions of the Ni matrix close to a CNT embedded. 

The reaction between Ni and CNTs forming Ni_3_C is improbable, due to the positive variation in the free energy of Giggs for this formation [36]. However, as it can be seen in Figure 7, the nanocomposites produced presented, although few, some nanometric and very localized particles of Ni_3_C carbides. The explanation for this can be related to some deformation that occurred during the processing of the powders, which potentiate this formation, or even with the possible presence of Ni particles inside the carbon nanotubes, due to their production, as mentioned in previous works [37,38,39,40,41]. The presence of the Ni_3_C phase, even in small quantities, can contribute to the strengthening effect of the nanocomposites since it can improve the bonding between the CNT and the matrix, as well as act as an obstacle to the movement of dislocation and grain boundaries. However, its contribution will be very small compared to other mechanisms such as load transfer.

**Figure 8:**
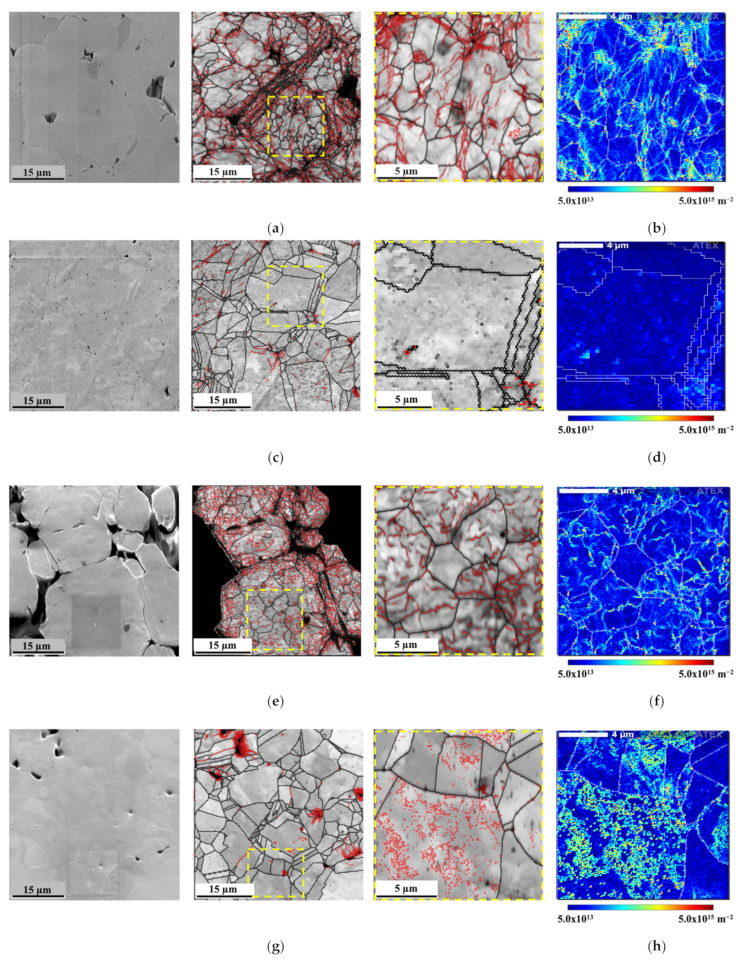
SEM images and IQ maps with high- and low-angle boundaries (black and red, respectively) delimited and geometrically necessary dislocation (GND) density maps for (**a**,**b**) Ni green compact, (**c**,**d**) sintered Ni, (**e**,**f**) Ni-CNT green compact, and (**g**,**h**) Ni-CNT sintered samples.

The increase in dislocation density due to the presence of CNTs in the metallic matrix can also promote the strengthening of the nanocomposites. When produced by powder metallurgy, there is an increase in dislocations observed during the different steps of production. Figure 8 presents the SEM images and IQ maps with high- and low-angle boundaries and geometrically necessary dislocation (GND) density maps of the green compact and sintered samples of Ni and Ni-CNT.

The green compact revealed a high density of dislocations, mainly on the surface of the Ni particles (as shown in Figure 8e with the low magnification), and in more deformed grains. The presence of CNTs in this processing step does not affect the microstructure.

**Figure 9:**
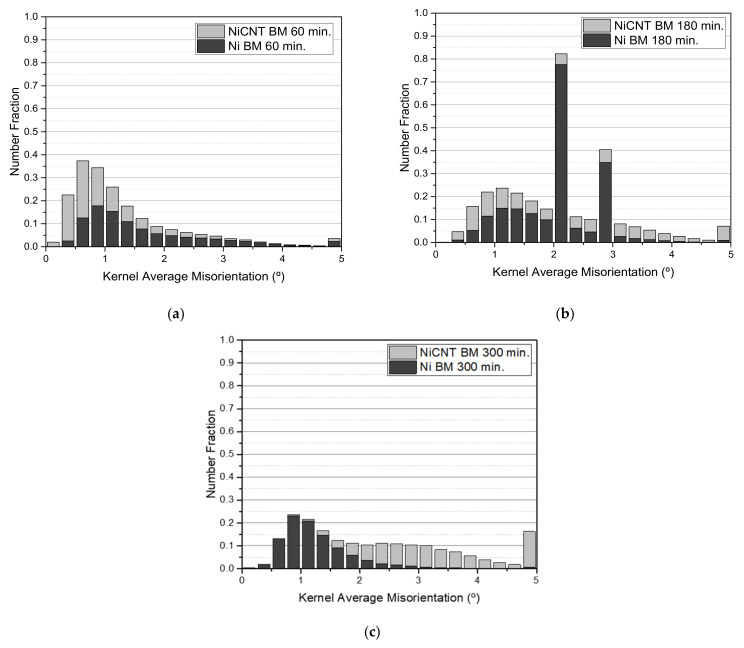
2nd neighbor KAM distribution graphs for the Ni and Ni-CNT samples produced by ball milling with times of (**a**) 60 min, (**b**) 180 min, and (**c**) 300 min.

The microstructure of the sintered samples revealed a decrease in the dislocation density during the sintering, since the activation of recovery and recrystallization mechanisms promotes the dislocation annihilation. In nanocomposites, a high estimated GND density (9.28 × 10^14^ m^−2^ against to 2.97 × 10^14^ m^−2^ for the Ni matrix) is observed mainly close to the CNT clusters. Both the presence of CNTs and second phase particles (although few) can influence the dislocation multiplication and interact with their motion and, consequently, affect the dislocation density of the nanocomposites. The presence of low-angle boundaries is related to the greater grain misorientation, being of the possible indicators of an increase in the dislocation density. Besides, the presence of twins and high-angle boundaries can reveal that the recovery and recrystallization processes occurred more efficiently. Previous work [29] has already shown that the presence of the CNTs significantly affects the recovery, recrystallization, and grain growth during the sintering of the nanocomposites. The density of dislocation of the Ni and Ni-CNT samples produced with different ball milling times was investigated through KAM results since it is one of the most adequate techniques to investigate the deformation remaining in the samples, directly related to the increase of dislocation density. Figure 9 shows the second neighbor KAM distribution graphs for the Ni and Ni-CNT samples produced using different ball milling times.

**Figure 10:**
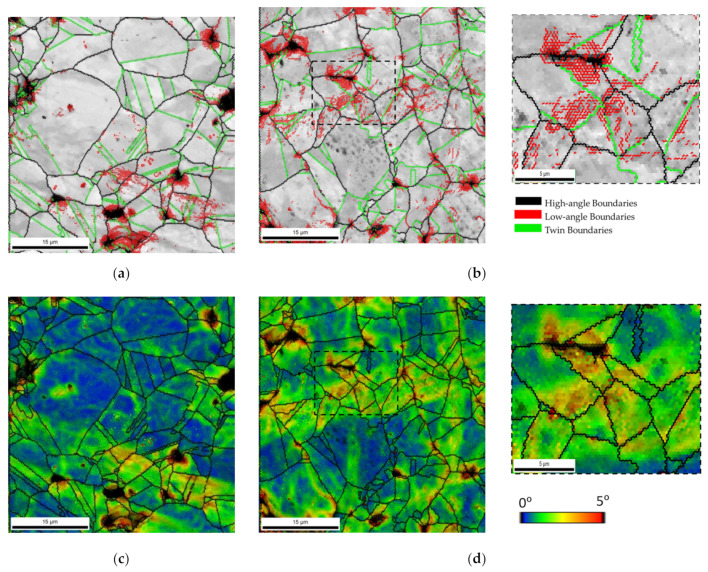
IQ maps with high- and low-angle boundaries (HAGBs and LAGBs) delimited, as well as the most common Ni twins (∑3 and ∑9) and 2nd neighbor KAM maps superimposed with IQ maps and grain boundaries delimited for the nanocomposites produced with (**a**,**c**) 60 min and (**b**,**d**) 180 min of ball milling.

For the samples produced with 60 and 180 min, the KAM distribution graphs are very similar, showing only a higher number fraction of grain misorientation for the nanocomposites. The nanocomposites produced with 300 min of ball milling reveal a local misorientation spread higher than the Ni samples produced under the same conditions, proving the significant effect of the CNTs on the dislocation arrangement, as well as on their motion and annihilation during the sintering. This effect is more notable for Ni-CNT produced using longer ball milling times since the respective dislocation density is related to the greater plastic deformation. KAM maps conducted a detailed characterization of the dislocation structure. Figure 10 shows the KAM maps for nanocomposites produced with ball milling times of 60 and 180 min. Based on the KAM maps, it is clear that the highest dislocation density is observed close to the CNT agglomerates. However, some grains apparently without CNTs clusters have high misorientation. This can be explained due to the presence of CNTs embedded in the Ni grains. The results indicated that the nanocomposites can be characterized by the presence of dislocation cells and subgrains, mainly those produced with 180 min of ball milling. The more uniform dispersion of the CNTs for the samples produced with 180 min of ball milling promotes a significant effect of the CNTs on the dislocation rearrangement.

**Figure 11:**
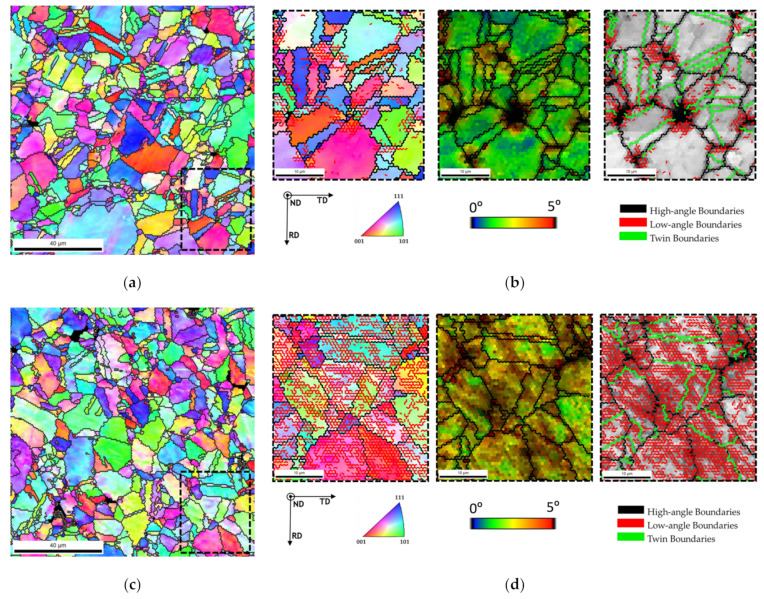
IPF maps of (**a**) Ni and (**c**) Ni-CNT produced by ultrasonication. IPF, KAM, and IQ maps with high- and low-angle boundaries (HAGBs and LAGBs) delimited by the (**b**) area marked in (**a**,**d**) area marked in (**c**).

The dispersion/mixture process has a significant effect on the increase of the hardness value that is related to the CNTs dispersion and with the CNT structure less damage. Figure 11 shows the IPF maps for Ni and Ni-CNT nanocomposites produced by ultrasonication. The higher magnifications reveal that the nanocomposites produced by this technique exhibit a higher low-angle boundary associated with a greater grain misorientation. KAM and IQ maps with high- and low-angle boundaries (HAGBs and LAGBs) proved that the grains apparently without pores and CNT clusters present a high density of low-angle grain boundary, as well as higher grain misorientation.

**Figure 12:**
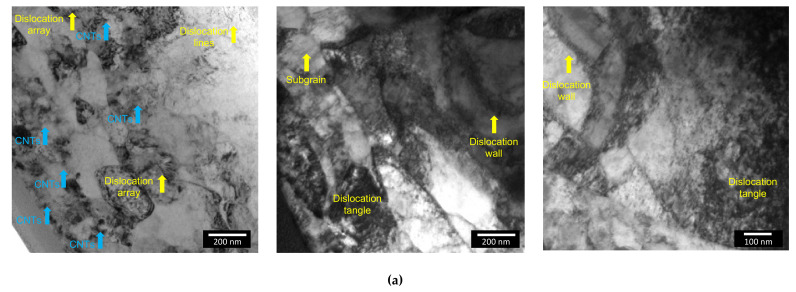

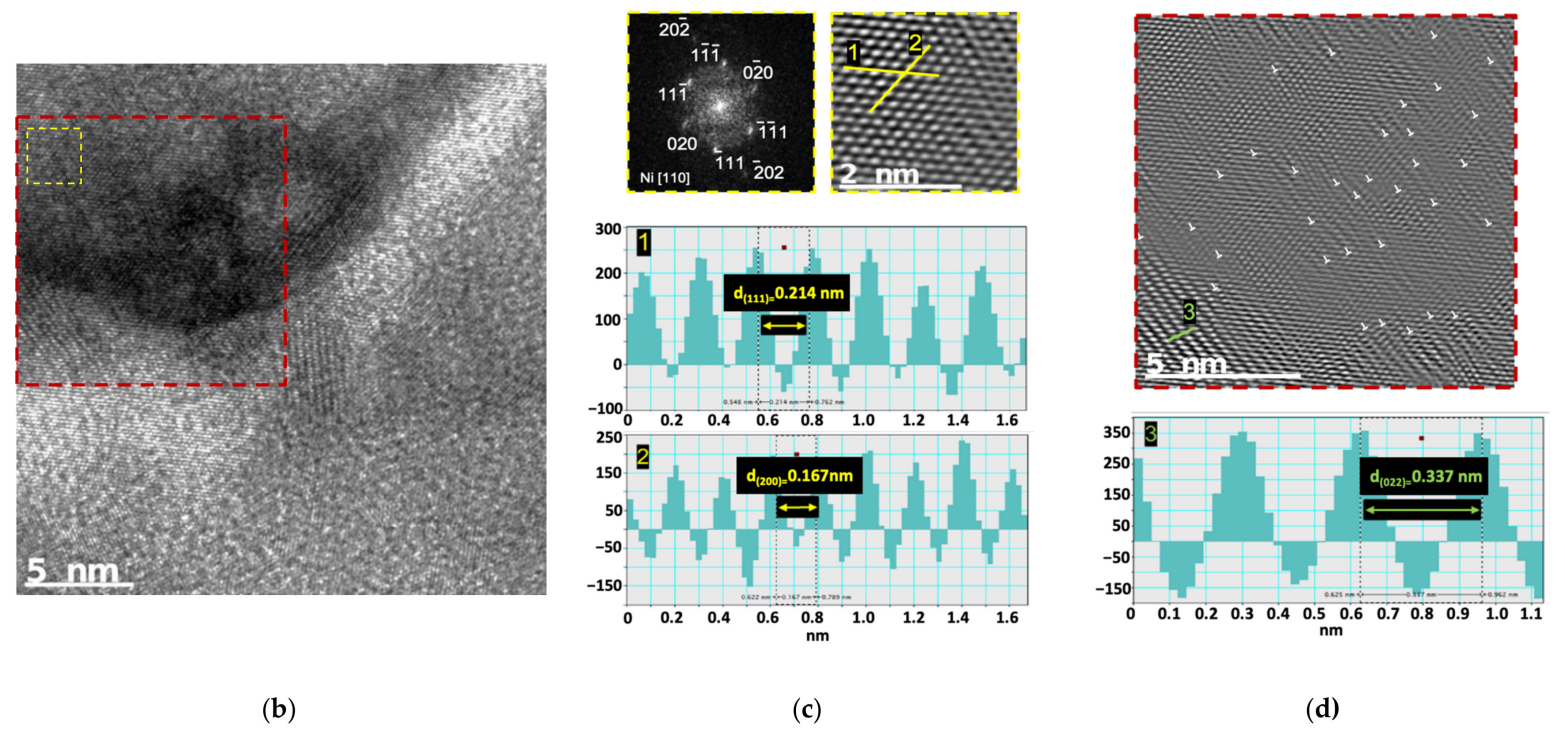
(**a**) TEM images showing dislocations and CNTs embedded, (**b**) HRTEM image of nanocomposite showing a region close to a CNT, (**c**) FFT, inverse FFT images and interplanar distance of the 1 and 2 lines profile, (**d**) inverse FFT of the marked red region showing the dislocations surrounding the CNT and interplanar distance of the 3 line profile.

Figure 12 shows TEM and HRTEM images of the nanocomposites produced by ultrasonication, revealing the presence of dislocation cell structure. TEM images revealed that a high density of the dislocation characterizes the nanocomposites. The nanocomposites microstructure is very different from the Ni produced under the same conditions [7]. HRTEM images showed that a high density of dislocations is observed close to the CNT embedded in the Ni matrix.

**Figure 13:**
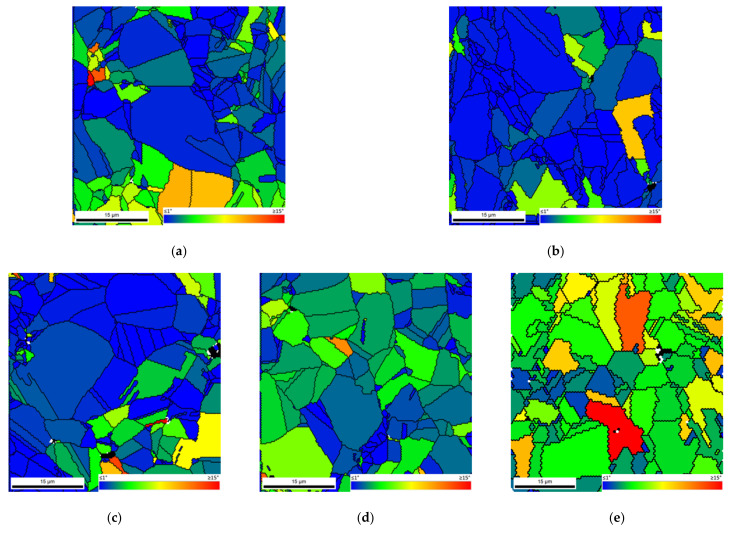
GOS maps of Ni produced by ball milling for (**a**) 60, and (**b**) 300 min and of Ni-CNT produced with (**c**) 60, (**d**) 180, and (**e**) 300 min of ball milling.

To understand the effect of the CNTs on the microstructure of the nanocomposites during sintering, GOS maps of the Ni and Ni-CNT produced with different ball milling times were obtained, as shown in Figure 13.

The GOS maps reveal a higher percentage of fully recrystallized grains (in blue) for the samples without reinforcement and for the nanocomposite ball milled for 60 min. The nanocomposites were produced using 180 and 300 min show a high percentage of mildly deformed grains (represented by the color green) and some significantly deformed (orange and red), especially in the Ni-CNT produced using 300 min of ball milling. These results indicated that CNTs may have an effect on the extension of the recovery, recrystallization, and grain growth processes during the sintering of the Ni matrix. This effect is more significant for the nanocomposites with a higher dislocation density (nanocomposite produced with 300 min of ball milling).

**Figure 14:**
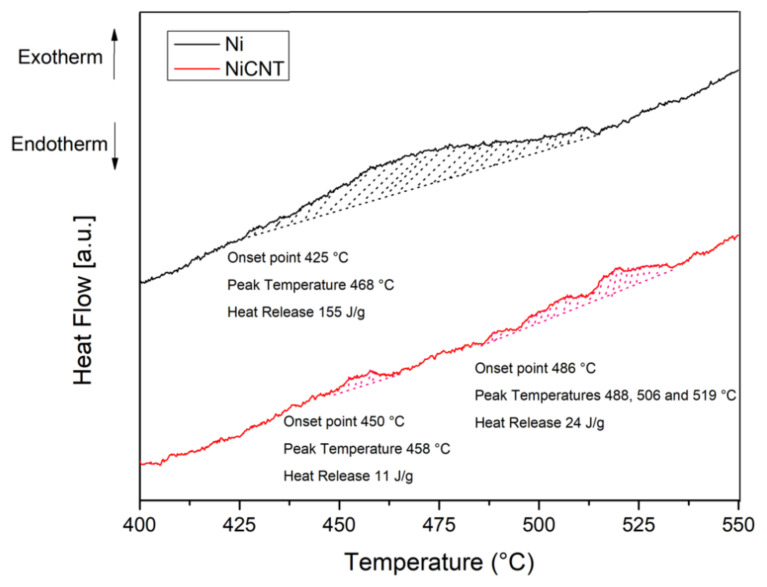
DSC curves of Ni and Ni-CNT produced with 300 min of ball milling.

Figure 14 displays the DSC curves of the Ni and Ni-CNT produced using 300 min of ball milling. For Ni sample, it is observed an exothermic peak with an onset point of 425 °C and with a heat release of 155 J/g. This exothermic reaction corresponds to the recrystallization of the sample. For the nanocomposite it is observed that this reaction occurs in different steps and with lower heat release. These results show that with the introduction of CNTs it is clear that the extent of recrystallization is affected.

**Figure 15:**
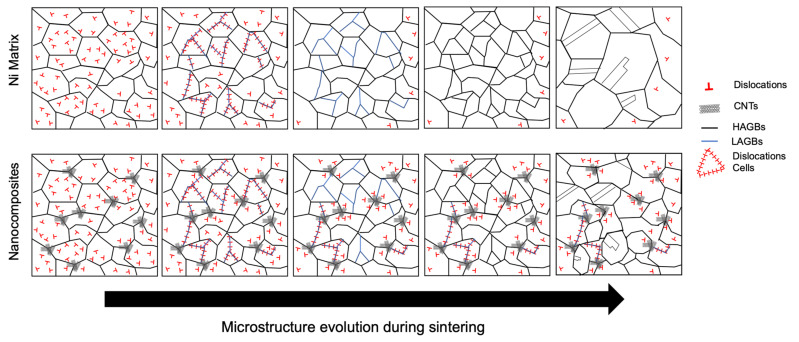
Schematic representation of the proposed effect of the CNTs on the microstructural evolution of the nanocomposites during sintering (HAGBs and LAGBs: high- and low-angle grain boundaries, respectively).

Based on the microstructural characterization, it is possible to present a viable mechanism about the effect of the CNTs on the Ni metal matrix, during sintering. Figure 15 shows a suggested microstructural evolution for Ni and Ni-CNT during sintering. In the beginning, the samples exhibited the presence of dislocations due to the dispersion/mixture processes used. During sintering, the dislocations are rearranged into low-angle grain boundaries with a misorientation of ≤5° (forming sub-grains) and some of them are annihilated. At this stage, the nanocomposites present notorious differences from the Ni matrix without CNTs, since their presence affects the rearrangement and annihilation of dislocations. Nanocomposites exhibit high dislocation density close to the CNTs which influence the rearrangement and formation of dislocation cells. During further sintering, the misorientation between sub-grain increases and, consequently, the sub-grains structures give rise to new grains. For the nanocomposite the misorientation evolution is affected and the modification of low-angle to high-angle grain boundaries is reduced. With the application of temperature over time, these grains grow, and twins are formed, although for the nanocomposite, the presence of CNTs acts as obstacles and hinders this process. The recrystallization of the nanocomposites does not occur to the same extent as the Ni matrix without CNTs, since the CNTs reduce the grain boundary mobility and dislocation density. The Orowan strengthening occurs when the reinforcement affects the dislocation motion, and geometrically necessary dislocation is induced due to the thermal mismatch between the matrix and the CNTs. The CNTs significantly affect the grain boundary migration during the sintering through Zener pinning. Therefore, the final microstructure of Ni is composed mainly of recrystallized grains, while the nanocomposites have dislocation cells, dislocation tangles, and the recrystallized grains present a smaller size, mainly in the vicinity of the CNTs.

## 4. Conclusions

The strengthening mechanisms of the Ni matrix nanocomposite reinforced with CNTs were investigated. The nanocomposites were produced by powder metallurgy using two different routes for the dispersion and mixing of the metallic powders with the reinforcement. The strengthening of the Ni matrix with the introduction of the CNTs was confirmed by the increase of the hardness values observed for all production conditions. Nevertheless, the dispersion of CNTs with a damage-free structure is essential for the effective strengthening of the Ni matrix. Load transfer is the mechanism that most contributes to the improvement of the mechanical properties of nanocomposites. Observations of the fracture surfaces proved that this mechanism is present in these nanocomposites. Grain refinement and the presence of second phase mechanisms have also been identified. However, the contribution of these mechanisms is reduced. The increase in dislocation density due to the presence of CNTs seems to play an important role in the strengthening mechanisms of these nanocomposites. The microstructure of sintered nanocomposites is characterized by less intense recrystallization and a higher density of dislocations than the Ni matrix without CNTs, due to the grain boundary pinning and strain hardening resulting from the CNTs presence.

## Data Availability

Data can be available upon request from the authors.
